# Supplementary material for: Rapid turnover of life-cycle-related genes in the brown algae
Source: Genome Biol. 2019 Feb 14;20:35. doi: 10.1186/s13059-019-1630-6 (PMC6374913; doi:10.1186/s13059-019-1630-6)
Supplement: Supplementary file 3 — Protein alignments, corresponding Gblocks html file and CDS sequences for the lineage conserved gametophyte-biased genes. (ZIP 2109 kb) [file 13059_2019_1630_MOESM3_ESM.zip › Con_GA_alignments/SJ19584.aa_ali.fasta-gb.htm]

SJ19584.aa.fasta.trans.aa\_ali.fasta


## Gblocks 0.91b Results

Processed file: **SJ19584.aa.fasta.trans.aa\_ali.fasta**  
Number of sequences: **2**  
Alignment assumed to be: **Protein**  
New number of positions: **179** (selected positions are underlined in blue)

```
                         10        20        30        40        50        60
                 =========+=========+=========+=========+=========+=========+
SJ19584          MSSVSSWPPTASWGSKVLQAMSHAAHAPKELGVVAGHARVLREWKQRWDRPGGKKQPLLQ
Mac.69040.1      MSSVSSWPPTASWGSKVLQAMSDAAHAPKELTVAAGHARVLREWKQRWDRPGGQRQPLLL
                 ############################################################


                         70        80        90       100       110       120
                 =========+=========+=========+=========+=========+=========+
SJ19584          QSRSEELIYSSSGKESGNVSTSDQDVSTANINNDGHNGGSGGGGGGGGGSGVGGGAGGGE
Mac.69040.1      QSRSEELIYSSSVKESGVVSNFDQDVSTAIANNTAHNNSSGSGANGGGG--IGGGDGGVE
                 #################################################   ########


                        130       140       150       160       170       180
                 =========+=========+=========+=========+=========+=========+
SJ19584          EEVSPMCLRCHYLTVNPSAPLPPFENADSATFFSSSSSSFSSGLAVDVTLGRYCLCSVNR
Mac.69040.1      EEISPMCLRCHYLTVNPSAPLPPFENVDSANAFSSSSSGF----PHDVTLGRYCLCSVNR
                 ########################################      ##############


                        190       200       210       220       230       240
                 =========+=========+=========+=========+=========+=========+
SJ19584          DIQREVLQGNLSSNSSSNPNPANPTTGDPPSSPSGNNNTNNNAEQGFHKNATASASSEGL
Mac.69040.1      DIQREVLQ---------------------------------------------VCT----
                 ########                                                    


                        250       260       270       280       290       300
                 =========+=========+=========+=========+=========+=========+
SJ19584          HSSHPRSPPLSGSGGAMAGDALHDAGSANTIFSLPSSPEGRGGGGGGEGGGGGEGGLISR
Mac.69040.1      ------------------------------------------------------------
                                                                             


                        310       320       330       340       350       360
                 =========+=========+=========+=========+=========+=========+
SJ19584          GGSGHAFGDPGSLSWATGTLPSGGGASGGTSGGTSGGTGGGGGGGIKRARDRRADRRRKK
Mac.69040.1      ------------------------------------------------------------
                                                                             


                        370       380       390       400       410       420
                 =========+=========+=========+=========+=========+=========+
SJ19584          DPFEALEERLLDGFSLESITRQLELQDDTLQKGVRHVERMEARREDRGRRSNDEHQRHLN
Mac.69040.1      ---------------------------------------------------------YIL
                                                                             


                        430       440       450
                 =========+=========+=========+
SJ19584          LQKKRLQAVTVASDPAAARKEERQSLLPPV
Mac.69040.1      L------------------YEE-------R
```

```
Parameters used
Minimum Number Of Sequences For A Conserved Position: 2
Minimum Number Of Sequences For A Flanking Position: 2
Maximum Number Of Contiguous Nonconserved Positions: 8
Minimum Length Of A Block: 10
Allowed Gap Positions: None
Use Similarity Matrices: Yes
```

```
Flank positions of the 3 selected block(s)
Flanks: [1  109]  [113  160]  [167  188]  

New number of positions in SJ19584.aa.fasta.trans.aa_ali.fasta-gb:  179  (39% of the original 450 positions)
```
